# Gene and cell therapy for children — New medicines, new challenges?^☆^

**DOI:** 10.1016/j.addr.2014.02.010

**Published:** 2014-06-30

**Authors:** Karen F. Buckland, H. Bobby Gaspar

**Affiliations:** a Centre for Immunodeficiency, Molecular Immunology Unit, UCL Institute of Child Health, London, United Kingdom; b Department of Clinical Immunology, Great Ormond Street Hospital NHS Foundation Trust, London, United Kingdom; c Cellular Therapies, Great Ormond Street Hospital NHS Foundation Trust, London, United Kingdom

**Keywords:** Gene therapy, Cell therapy, ATMP, Stem cell, Translational, Vector, GMP, Biologics, Autologous

## Abstract

The range of possible gene and cell therapy applications is expanding at an extremely rapid rate and advanced therapy medicinal products (ATMPs) are currently the hottest topic in novel medicines, particularly for inherited diseases. Paediatric patients stand to gain enormously from these novel therapies as it now seems plausible to develop a gene or cell therapy for a vast number of inherited diseases.

There are a wide variety of potential gene and cell therapies in various stages of development. Patients who received first gene therapy treatments for primary immune deficiencies (PIDs) are reaching 10 and 15 years post-treatment, with robust and sustained immune recovery. Cell therapy clinical trials are underway for a variety of tissues including corneal, retinal and muscle repair and islet cell transplantation. Various cell therapy approaches are also being trialled to enhance the safety of bone marrow transplants, which should improve survival rates in childhood cancers and PIDs. Progress in genetic engineering of lymphocyte populations to target and kill cancerous cells is also described. If successful these ATMPs may enhance or replace the existing chemo-ablative therapy for several paediatric cancers. Emerging applications of gene therapy now include skin and neurological disorders such as epidermolysis bullosa, epilepsy and leukodystrophy. Gene therapy trials for haemophilia, muscular dystrophy and a range of metabolic disorders are underway. There is a vast array of potential advanced therapy medicinal products (ATMPs), and these are likely to be more cost effective than existing medicines. However, the first clinical trials have not been without setbacks and some of the key adverse events are discussed. Furthermore, the arrival of this novel class of therapies brings many new challenges for the healthcare industry. We present a summary of the key non-clinical factors required for successful delivery of these potential treatments. Technological advances are needed in vector design, raw material manufacture, cell culture and transduction methodology, and particularly in making all these technologies readily scalable.

## Graphical abstract

**Figure:**
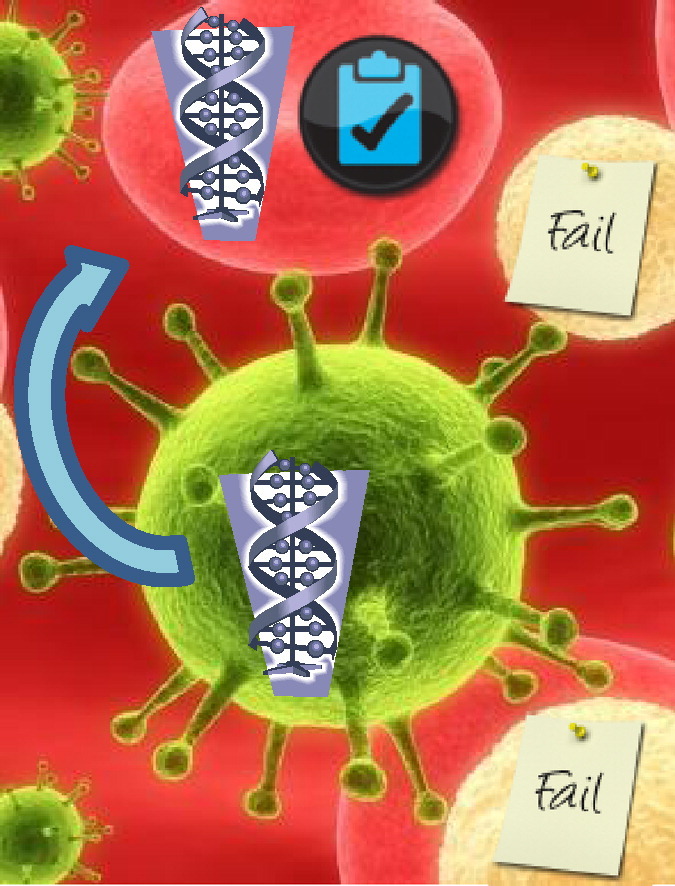

## 1 Introduction and definitions

### 1.1 Introduction

**Fig. 1:**
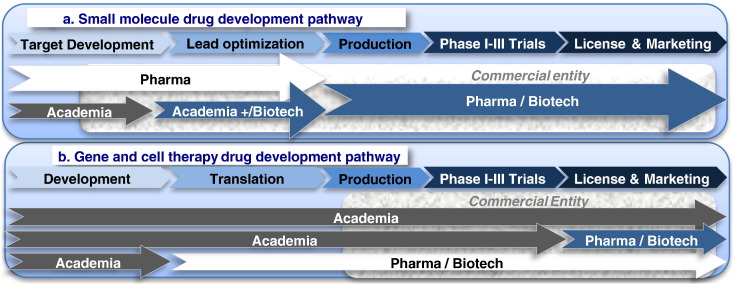
a. Small molecule drug development pathway. b. Gene and cell therapy drug development pathway.

We are currently undergoing a technological revolution in medicine. The elucidation of the human genome and the development of high throughput gene sequencing technologies have led to the rapid understanding of the genetic basis of many inherited diseases. We have also seen remarkable technological advances in gene transfer and cell manipulation and culture, which enable specific engineering of both human stem cells and terminally differentiated cell populations for many applications. These developments are leading to new therapeutic options for a wide range of inherited and acquired conditions and have challenged our conventional view of ‘medicines’. However, at present, both cell and gene modified cell therapies have almost exclusively been developed and used in the academic environment. Their translation is presently occurring in basic research centres, under compassionate use schemes or in approved hospital-based clinical trials (see Fig. 1). The co-localisation of clinicians, patients and scientists where partnerships exist between a university and a hospital has been fundamental especially in rare diseases to moving directly to testing in patient groups (Phase I/II clinical trials) without first testing in healthy human volunteers (Phase I clinical trials). However as we approach 20 years since the first clinical trials the question arises as to whether this seclusion in the academic environment is now restricting growth. The focus of this review is to describe the status of development of cell and gene therapies and the path to translation outside of the academic realm and into licenced, marketable medicines.

### 1.2 Definitions

#### 1.2.1 ATMPs

At present in the EU, cell and gene therapies are regulated under the guidelines for advanced therapy medicinal products abbreviated to ‘ATMPs’. There are three categories of ATMPs, i) somatic cell therapy, ii) gene modified cells and iii) xenotransplantation. The distinctions are subtle, and another popular term ‘stem cell therapy’ is often used to describe treatments that fall into either category. Occasionally treatments could fall into more than one category. For instance xenotransplantation of isolated cells is moving forward with the use of porcine pancreatic islet cells for the treatment of type I diabetes [1]. In this review we will focus upon the status of somatic cell therapy and the use of gene modified cells, and aim to organise them by the ATMP categorisation.

#### 1.2.2 Stem cell therapy

Stem cell therapies can be divided into those involving gene modified cells and those not. For example: A CD34 + cell selection from bone marrow is regarded as a haematopoietic stem cell transplantation (HSCT) as these cells are not significantly altered from their original state and as such does not require an ATMP manufacturing licence. If, however, CD34 + cells from the same source are cultured with e.g. cytokines, activating antibodies or a gene therapy vector, they become modified CD34 + cells and are regarded as an ATMP. The use of the term ‘gene therapy’ should be applied only to an ATMP where a gene delivery vehicle or vector is employed to generate gene modified cells.

#### 1.2.3 Cell therapy

A *cell therapy* ATMP is a somatic cell therapy that goes beyond the definition of a transplant but does not involve genetic modification of cells. A treatment becomes a cell therapy (and an ATMP), when the preparation involves modification or expansion of the cells in culture. A simple selection or enrichment of a particular group of cells from a tissue does not make it an ATMP. There are now examples of stem cell therapy, gene therapy and somatic cell therapy developments for a wide range of tissues.

### 1.3 Development pathways

The progression from research and development to market of a cell or gene therapy ATMP is not likely to follow the traditional path of a small molecule medicine (Fig. 1a). Academic involvement continues long after the therapy becomes a commercial entity, and in some cases an academic consortium may take a product all the way to market (Fig. 1b). For rare diseases, this is enabled by the orphan drug designation (USA) or orphan designation (within the EU) a separate route created to incentivise development of medicines for rare diseases by the FDA and EMEA. A similar programme exists also in Japan.

## 2 Cell therapy for paediatric disease

In the UK there were 34 somatic cell therapy clinical trials open to recruitment in the first quarter of 2013 [2]. Here we shall introduce the key developments for conditions affecting children.

### 2.1 Corneal and retinal repair

Researchers have been decoding the developmental and differentiation pathway that takes embryonic stem cells (ESCs) to the functional cell types of the eye. The aim is to use donor ESCs to replace defunct areas of the retina and in order to improve vision in forms of blindness involving diseased retinal tissue [3]. ESCs from the morula were chosen as the starting cell material for cell therapy of diseased retina as they are totipotent, that is able to differentiate into any adult cell type, and have potentially unlimited self-renewal capacity. Transplantation of human ESC-derived retinal pigmented epithelial cells was pioneered by ophthalmologists Schwartz and colleagues based at University of California, Los Angeles, USA. The first two patients treated were adults with two different forms of macular degeneration, Stargardt's disease (an inherited form of juvenile macular degeneration which affects ~ 1/10,000 children) and dry age-related macular degeneration (AMD) [4]. Retinal pigment epithelial (RPE) cells derived from embryonic stem cells are now being grown by Advanced Cell Technology (ACT) as a cell therapy for Stargardt's disease. This novel cell therapy is currently being tested in multi-centre dose finding Phase I/II clinical trials in the Jules Stein Eye Institute, USA and at London's Moorfields Eye Institute. The FDA has awarded ACT's RPE cells orphan drug designation, which will help to make this treatment more readily available for other patients upon completion of clinical trials.

Chemical and other types of burn injuries to the eye can result in corneal destruction and are associated with limbal stem-cell deficiency. Researchers in Milan have developed a method for ex vivo expansion culture of autologous limbal stem cells explanted from a healthy eye to create a larger curative patch for the treatment of limbal stem-cell deficiency in a damaged eye [5]. The 112 patients enrolled included some children (age range: 14–80, median: 46.5 ± 14.4 years). This cell therapy requires a 1.5 × 1.5 nm explant of healthy tissue from the limbal area to be cultured for 2–3 weeks before transplantation of the limbal stem cell graft into an affected area. A Phase II clinical trial is also underway in Newcastle, UK where 7 subjects have been successfully treated to date and orphan drug designation is sought.

Other ocular cell therapy treatments in earlier stages of development largely at Moorfields Eye Hospital NHS Foundation Trust and elsewhere include photoreceptor generation from both embryonic and iPS stem cells [6]. These are expected to impact a wide range of inherited blindness conditions.

### 2.2 Pancreatic islet cell therapy

There are approximately 25,000 young persons with diabetes in the UK and approx. 150,000 in the USA. Diabetes is typically diagnosed between 10 and 14 years of age, and prevalence of both type I and type II in children is rising. Approximately 10% of type I diabetes mellitus (T1DM) patients are so exquisitely sensitive to insulin that injectable replacements do not provide adequate or optimal management of their blood sugar levels. Islet cell therapy is proposed as an alternative option for this subset of patients. Cell therapy for type I diabetes received proof of concept with the first successful pancreatic islet transplantation in rats in 1972 and humans in 1990 [7,8]. By 2000 Shapiro and colleagues in Edmonton, Canada had developed an optimised cell therapy that uses a larger dose of islet cells in conjunction with a non-glucocorticoid immune suppression, consisting of anti-CD25 mAb induction, sirolimus and low-dose tacrolimus [9]. The Edmonton protocol has now been tested in at least 10 centres around the world and has dramatically improved the insulin-independence of subjects receiving pancreatic islet cell transplantation. Subsequent advances made in islet cell isolation, digestion, culture and transportation strategies, which improve both yield and longevity ex vivo and function after transplantation have improved the 1 and 3 year outcomes. However, long term sustainability > 5 years of insulin production has not yet been achieved, likely due to acute and chronic rejection, recurrence of autoimmunity and ongoing pharmacological damage to the graft [10].

### 2.3 Muscle repair

Both autologous transplantation and allo-transplantation of myoblasts are in development for a number of conditions including cardiac infarction, urinal and anal incontinence and muscular dystrophy. New myoblast-based strategies for the treatment of muscular dystrophy including DMD are of particular relevance for children. X-linked Duchenne muscular dystrophy (DMD) is one of the most common and severe muscular dystrophies with symptoms starting in early childhood. Phase I/II clinical trials of cell therapy using CD34 + or CD133 + autologous stem cells are beginning to show improvement of muscle function in DMD [11]. Allo-transplantation of human umbilical cord blood-derived mononuclear cells is also in Phase I/II trials for the treatment of hereditary ataxia [12]. This is a particularly interesting venture as the therapy is being developed with funding from the Chinese government at Shenhen Beike Biotechnology, China. Beike Bio. has the most comprehensive stem cell bank in Asia and extensive GMP ATMP stem cell facilities thus it could become the first to manufacture ATMPs at a sizable quantity. We will address ATMP manufacturing quantities later.

### 2.4 Haematopoietic stem cell therapy adjuncts

Haematopoietic stem cell transplant (HSCT) is the mainstay of treatment for a wide range of childhood diseases. However patients receiving partially matched transplants are at great risk of developing graft vs. host disease (GvHD). Patients are also at high risk of infection in the first 6 months following HSCT, prior to immune reconstitution by the engrafted cells. Furthermore, previous viral infections such as CMV may reactivate during the first few months in an immunocompromised post-engraftment individual. Hence a number of gene and cell therapies are in development to increase the safety of HSCT. These include both enrichments and depletion of specific cell subsets. CD3 T cell depletion can be employed to reduce GvHD but at the expense of anti-viral immunity. Thus Amrolia and colleagues at Baylor College of Medicine, USA and Great Ormond Street Hospital have developed a method for the depletion of allo-reactive T cells from a HSCT before transplantation. Initial studies had promising results with 16 patients treated. A second clinical trial with a further refined allo-depletion method is expected to open to recruitment in January 2014 [13,14]. Researchers at King's College London & Guy's & St. Thomas NHS Foundation Trust are also aiming to reduce incidence of GvHD by an alternate method employing in vitro expanded regulatory T cells. Their ‘NTREAT’ trial is also expected to open soon [15]. Alternatively a CD3 depleted HSCT can be supplemented by adding back only viral-specific T cells. This so called adoptive transfer of antigen-specific donor T cells can be employed as either a prophylactic or therapeutic treatment and has been successful in reconstitution of immune responses to EBV, CMV and adenoviruses in a number of clinical trials [16–18]. Genetic modification of cells can be employed to generate antigen-presenting cell lines as an alternative to antigen or peptide activation, or to further enhance this therapeutic by generating cytotoxic T lymphocytes with specificity for multiple viruses in a single culture [19,20]. Generation of viral-specific T cells on a per patient and typically reactive basis is costly and in some cases may be too slow, thus a ‘cell bank’ approach has been proposed and its feasibility is being explored [21]. In a recent multicentre trial, frozen third party virus-specific T cells from donors with common HLA types were used to treat 50 patients with severe EBV, CMV or adenoviral infections post-HSCT. The results were encouraging with partial or complete viral responses in 74% of patients, no immediate SAEs and only 2 incidences of novel GvHD [22].

## 3 Gene therapy for paediatric disease

A gene therapy treatment may be ex vivo, in situ or in vivo. Ex vivo is the route taken predominantly for gene modification of bone marrow-derived cells and epidermal sheets. In vivo gene therapy is when the gene delivery vehicle is administered directly to the patient. In vivo gene therapy might also be described as in situ e.g. inhalation of a gene therapy vector, which is proposed for the modification of lung epithelium. The gene delivery vehicle is normally directed at a specific cell target. The route of administration is dictated by the location and accessibility of the target cells. The first gene therapy treatment to receive approval, alipogene tiparvovec (Glybera®) is delivered as a series of intramuscular injections. Clinical trials are also underway with intravenous injection of a gene therapy vector for haemophilia. The majority of gene therapy products trialled to date are ex vivo ATMPs, and of these by far the majority are haematopoietic stem cell products but the advances made with HSCs are starting to be applied elsewhere.

### 3.1 Ex vivo gene therapy treatments

#### 3.1.1 Haematopoietic stem cells (HSCs)

For this type of treatment autologous CD34 + cells are selected from the bone marrow or an apheresis product using standard HSCT selection procedures. The selected cells are then cultured in a laboratory in a defined cell culture medium containing a cocktail of cytokines. The gene therapy vector is added to the culture whereupon a copy of the therapeutic gene is introduced to the genome of the target cells. The ‘gene-corrected’ cells are then re-administered to the patient in the form of an ‘autologous gene modified CD34 + cell’ transplant. Great advances have been made in the treatment of primary immune deficiencies and metabolic disorders with gene therapy including X-linked severe combined immunodeficiency (SCID), adenosine deaminase deficiency (ADA), Wiskott-Aldrich syndrome (WAS) and chronic granulomatous disease (CGD) [23,24]. The breadth of application of this technology is expanding to include other types of hereditary conditions e.g. Fanconi anaemia, childhood cerebral adrenoleukodystrophy (CCALD), metachromatic leukodystrophies (MLDs) and X-linked lymphoproliferative syndrome (XLP).

The first clinical trials for gene therapy of primary immune deficiencies used gamma retroviral (γRV) vectors. These vectors are particularly suited to the transduction of haematopoietic cells as their structure is derived from the genera of viruses that include HIV. Sustained immune reconstitution was achieved with > 10 year persistence of gene corrected cells. Incidence of insertional mutagenesis through activation of proto-oncogenes in the first clinical trials for gene therapy of X-linked SCID was an early setback for the technology. However, the successful disease correction and the lack of other side effects in the majority of patients in these early trials have driven forward development of ever safer vectors. One such improvement was the design of self-inactivating (SIN) vectors which have a reduced potential for insertional mutagenesis as a result of deletions in viral promoter elements.

The translation of gene therapy using γRV vectors was also hampered by their low titre (typically 1–5 × 10^6^ Ig/ml for GMP grade) and dependence on actively dividing cells for effective integration. Transduction protocols using γRV are laborious requiring pre-stimulation of cells to initiate cell division, pre-loading of vector onto the surface of cell culture containers, and multiple rounds of transduction. Improved vectors using a lentiviral (LV) backbone were introduced to circumvent these issues. LV vectors can be produced at much higher titre (in 2012 GMP grade LV vectors were typically supplied in the 10^8^–10^9^ Ig/ml range) and enable streamlined transduction protocols. The latter further improves safety as the decreased level of manipulation required lowers the risk of microbiological contamination, and in the case of CD34 + stem cells, allows preservation of multi-potency. LV vectors also display a safer integration profile than their γRV counterparts and demonstrate robust gene expression in a range of cell types [25].

Increased safety expected of SIN design vectors derived from either γRV or LV has allowed the approval of further Phase I/II clinical trials for X-linked SCID (SIN γRV), or SIN LV protocols for WAS, ADA and CGD. The future may lie in alpha retroviral (αRV) vectors, which have been shown to have a propensity for insertion in extra-genic regions and would therefore be expected to have lower genotoxicity [26]. SIN αRV vectors have been described and clinical trials are anticipated for CGD [27].

ADA gene therapy appears to be the first of the ex-vivo gene therapy applications that is likely to make the leap into routine therapy, with a γRV programme in development by GlaxoSmithKline in conjunction with researchers in Milan. A Phase I/II trial for ADA with a LV vector has recently opened in the UK & the USA and an accompanying orphan drug designation application has been granted by MHRA.

#### 3.1.2 Mature T cells

There are a number of therapies in development which use gene therapy vectors to generate gene modified T cells. The early T cell based gene therapy trials have used γRV vectors but a number of approved trials using LV vectors are currently underway. Gene modified T cells are proposed for the treatment of cancer in several ways: a) Generation of T cells to directly target tumour cells or b) enhancing the safety of an allogenic bone marrow transplant (BMT).

There are two major strategies for generating tumour-specific T cells, i) using gene therapy to generate T cells with chimeric antigen receptors (CARs) or redirected T cell receptors (TCRs) or ii) expansion of tumour infiltrating lymphocytes (TIL). Autologous genetically modified CD19 or CD30 CAR + T cells are in trials for paediatric ALL and CLL, WT-1 and HBV + tumours. The success of early studies has led to a partnership between the University of Pennsylvania and Novartis for the clinical development of CARs, which may speed their progress to market. However application to childhood diseases in the US will be hampered by the requirement to initially demonstrate efficacy in adults. With regard to technological progress Carl June, University of Pennsylvania, speaking at the ESGCT Congress 2012, described progress in the field as ‘using CARs we can eliminate tumours but it is not yet known how to eradicate them [tumours] and prevent relapses’. Early clinical trials show TIL to be effective for melanoma, but attempts to treat cancers more commonly occurring in children such as osteosarcoma and neuroblastoma have so far been unsuccessful, possibly due to an anergic cytokine environment within these tumours [28]. Recent reports of serious adverse events involving a ‘cytokine storm’ suggest the need for caution in this area with regard to the cell dose administered and the inclusion of co-stimulatory signalling domains [29–31].

In the context of improving the safety of allogeneic HSCT, a strategy using genetic modification of donor T cells to confer susceptibility to ganciclovir has been used. The inclusion of a ‘suicide gene’ allows the engrafted cells to be selectively targeted and deleted in the host in cases of intractable GvHD. Alternatively EBV-specific, CMV-specific and/or adenovirus-specific T cells can be specifically engineered or selected and reintroduced alongside a CD3-depeleted HSCT to enhance anti-viral responses following chemotherapy and BMT. Clinical trials using CMV-TCR modified allo-T cells are underway at UCL.

#### 3.1.3 Skin cells

Disruptions in the genes for keratin, collagen and laminin cause rare but devastating skin disorders with significant infant mortality. Colleagues at Great Ormond Street Hospital and Guys and St. Thomas's Hospital NHS Trusts have taken the knowledge gained with HSC and T cells and are moving forward directly with LV modification in design of clinical trials for Netherton syndrome (NS) and epidermolysis bullosa (EB). Proof of concept was achieved with successful engraftment of gene corrected keratinocytes in an adult patient with junctional epidermolysis bullosa [32]. A Phase I/II trial of gene therapy for Netherton syndrome open to children and adults is expected to start recruiting patients in the 1st quarter of 2014, and a second trial for epidermolysis bullosa will open later in the year. The major challenge for gene therapy of skin sheets is the sheer size of the organ to be replaced. It is hoped that the introduction of skin patches secreting the normal protein will allow spread of the secreted protein beyond the transplanted region to allow recovery of barrier function to larger areas of the body [33].

### 3.2 In vivo gene therapy treatments

Adeno-associated viral (AAV) vectors have been predominantly used as in vivo gene therapy vehicles due to their low immunogenicity combined with a breadth of viral tropism that allows for entry into a variety of target cells. Like LV vectors, AAV can enter both dividing and quiescent cells. Pseudotyping is used to fine tune the infectivity of AAV vectors.

#### 3.2.1 Metabolic disorders

Glybera® is the first gene therapy treatment to receive an EC marketing authorisation, and the first gene therapy to be approved anywhere in the world. Glybera is a treatment for lipoprotein lipase (LPL) deficiency (LPLD) an inherited condition with an incidence of ~ 1/500,000 births. The majority of LPLD cases are only diagnosed in adulthood but symptoms are often present from the first months of life. Methods to diagnose LPLD are improving and a trend towards earlier diagnosis is expected. The LPL gene is packaged in an AAV vector which is administered by intramuscular injection. Efficacy has been demonstrated in 3 clinical trials with long-term expression of biologically active lipoprotein lipase and a reduction in frequency of pancreatitis. Long-term follow-up is ongoing but so far all 3 trials have had a good safety profile [34].

Other metabolic disorders have now been targeted successfully in early phase clinical trials using HSC gene therapy with lentiviral vector mediated gene transfer. Adrenoleukodystrophy (ALD) is a group of progressive neuropathies. Childhood cerebral ALD accounts for about 1/3 of ALD cases and without treatment results in a vegetative state in early childhood. BMT can be effective for this disease but carries its own risks. Metachromatic leukodystrophy (MLD) is similarly devastating. Without treatment children with the infantile form will die by age 5, whilst those with juvenile MLD may survive to their early 20s or 30s. Gene therapies for ALD and MLD are in Phase I/II trials; whilst gene therapy for mucopolysaccharidoses (MPS) is currently in the preclinical stage [35].

Intra-cranial delivery using AAV has reached clinical trials for another leukodystrophy known as Canavans disease and also by this route preclinical development with lentiviral vector for the treatment of focal neocortical epilepsy is underway [36–38]. With over 40 known leukodystrophies we can expect many more clinical trials.

#### 3.2.2 Childhood blindness

In vivo application of gene therapy is ideally suited to immune privileged organs such as the eye. Vectors for optical conditions are typically designed on an AAV2/2 or more recently AAV2/8 backbone. Leber congenital amaurosis (LCA) a common form of retinal dystrophy is thought to account for 1/5 of cases of childhood blindness. Researchers at the Moorfields Eye Institute in London have a number of therapies in the pipeline for LCA caused by mutations in retinal genes including RPE65, AIPL1, RDH12 and RetGC-1. Gene therapies for achromatopsia and X-linked retinitis pigmentosa (RP) are also in development. For RP due to Prph2 gene a therapy is in development using rAAV to deliver RNAi silencing of the damaged gene [39]. Several patients with LCA caused by mutations in RPE65 have been enrolled in a Phase I/II study. Of 3 subjects receiving a single intraocular injection of the rAAV 2/2-hRPE65p.hRPE65, one has showed an impressive improvement in visual acuity in low light conditions [40]. Other centres are developing gene therapy for age-related macular degeneration, uveitis, choroideremia and diabetic eye disease. With treatments already in progress for retinal, corneal and macular dysfunction it is likely that many structures within the eye can one day be treated with some form of cell therapy. Together these form a remarkable leap forward in the prognosis for inherited and acquired causes of blindness.

#### 3.2.3 Haemophilia

Gene therapy has been proposed as a treatment for haemophilia for many years. The first attempts to treat either haemophilia A or B either failed to generate persistent protein expression and had immune-mediated toxicity or had only transient protein expression due to immune-mediated deletion of transduced cells [41]. A number of strategies to refine gene therapy for haemophilia are currently being investigated notably a switch to the use of AAV8 serotype vectors, and subsequent modifications to vector design such as i) increasing the specific activity of the factor IX (FIX) expressed by introduction of favourable sequence variants and ii) improved manufacture methods to reduce the level of empty capsids and hence the immunogenic load. There are currently 3 actively recruiting trials of gene therapy for FIX listed on clinicaltrials.gov. Early reports from one of these, a multicentre gene therapy trial using an AAV for the expression of factor IX for haemophilia B, recruiting at University College London Hospital, Stanford Medical School and St. Jude Children's Research Hospital are reporting great success in adults. If sufficient safety and efficacy are demonstrated, we can expect recruitment to be expanded to include juveniles in the near future and further trials specifically for children [42] and personal communication. Successes in gene-mediated correction of factor IX deficiency can be expected to accelerate development of strategies for factor VIII and haemophilia A.

### 3.3 Non-viral vector delivery systems

#### 3.3.1 Respiratory disorders

Cystic fibrosis (CF) is a severe, life limiting condition, characterised by viscous secretions, scarring and cysts in the lungs, pancreas, liver and intestines. Most cases manifest in the first year of life and CF has an incidence of 1:2,000–15,000 worldwide. The causative CFTR gene was identified in the 1980s and disease causing mutations are carried by approx. 1/30 of the western population. The challenge in targeting CFTR mutations is in their location. The affected cells are structural so BMT is not applicable. Gene therapy to treat the respiratory aspects of the disease with an inhaled vector is appealing but any gene therapy agent will require repeated applications in a mucous-filled hostile environment. Viral vectors are not suitable for repeated application thus a CFTR-expressing plasmid complexed with a cationic lipid ‘nanoparticle’ has been developed and is currently being tested in clinical trials. Over 70 patients have now received at least one dose of gene therapy in the ‘Wave I’ gene therapy of cystic fibrosis a Phase IIB trial being conducted at 2 sites in the UK [43].

#### 3.3.2 Muscular dystrophies

A number of gene therapy approaches are in development for Duchenne muscular dystrophy (DMD). There are two notable commercial enterprises one led by GSK the other by Sarepta. Both are employing anti-sense oligonucleotides to target exon 51 and both have progressed to Phase IIB–III clinical trials. Development of therapies targeting other exons, particularly exon 53, are still needed to make therapy available for all types of DMD. Improved methods of tissue targeting are needed to restore dystrophin in all tissues and may be provided by peptide-conjugated oligonucleotides. The progress of gene therapy development for DMD has been comprehensively reviewed by Benedetti [44].

## 4 Driving gene and cell therapy medicines forward

### 4.1 The ATMP development pathway

The progression from R&D to clinical trial is expedited for cell and gene therapies compared to traditional medicines i.e. small molecule, antibodies and vaccines. ATMPs are often personalised medicines where Phase I safety testing in healthy human volunteers is neither appropriate nor relevant. Furthermore the randomised double-blinded placebo-controlled Phase III trial, the benchmark of traditional small molecule development, is not feasible for ATMPs especially as many are in the field of rare diseases. One cannot have a placebo for a skin graft or a stem cell transplant. There are too few patients to randomise into different treatment arms, and ‘blinding’ of participants and their clinicians is impractical if not impossible. Thus many of the clinical trials reported are Phase I/II studies where proof of both efficacy and safety is sought. From a successful Phase II study, the possibility exists for progression to licensing and marketing authorisation. However, this next phase is hampered by the availability of cell culture facilities and in most cases the lack of technology to manufacture at scale sufficient for national or international supply. A number of technological advances are desperately needed to advance the whole field towards large scale, multi-national distribution (summarised in Table 1). To date, although several have received orphan drug designation only 4 ATMPs have received marketing authorisations: Glybera, Epicel, Carticel and ChrondroCelect. Despite the 20 years of clinical trials there is only one licenced gene therapy treatment, Glybera. The slow rate of progress has been acknowledged and a tide change is forecast. Political objections to stem cell research in the USA have declined in recent years. In 2012 a UK government funded body, called cell therapy catapult, was established with the aim to accelerate commercialisation of cell therapy technology.

It seems that a gene or cell therapy ‘fix’ can now be envisioned for a large number of inherited and acquired disease. However, for this vision to reach fruition the business model for manufacture of ATMPs has to be different from traditional pharmaceutical manufacturing. Cell products have short expiry and require unusual transport conditions compared to a traditional medicine. Batch manufacture is often replaced by independent manufacture runs of a single personalised product and with this comes significant individual variation of each preparation. The key to rapid translation from research to therapy is an understanding of the GMP process from the beginning (see Table 2), and as ever, access to appropriate resources (summarised in Tables 3 and 4). At present the field remains at a very early stage and the models for true commercialisation are yet to be established.

### 4.2 Cost vs. benefit?

In the majority of cases cell and gene therapies represent good value compared to conventional treatments. For instance, in the UK limbal stem cell therapy costs an average of £3,000/subject whereas a conventional corneal transplant is billed at £6,000. The latter would also require more significant aftercare to reduce and GvHD, thus an autologous limbal stem cell therapy has potential to be doubly economical. Likewise an allogeneic bone marrow transplant for a patient with a primary immunodeficiency costs typically £250,000 and upwards, given the cost of inpatient stay, drugs and frequency of complications such as GvHD. In contrast, manufacture of autologous gene modified CD34 + cells can be achieved for approx. £15,000–30,000. The additional follow-up costs of gene therapy treatment for primary immune deficiencies are likely to be significantly less given that the length of hospital stay, long-term prophylactic medication requirements and other post-therapy complications are considerably reduced compared to conventional BMT. GMP manufacturing costs for the ATMPs currently in clinical trials at Great Ormond Street Hospital are broken down into vector procurement costs of £5,000–15,000 per trial participant, largely dependent on the individual's age/body mass; plus aseptic manufacture costs typically £10,000 ± £3,000 per patient per product, the range being dependent upon the number of days of culture required and the complexity of the gene transfer process.

The extreme specialisation of cell and gene therapies brings many unusual and often costly implications. A unique challenge in rare genetic disorders is in the youth of the paediatric patient. National and private healthcare rarely provides for the needs of the accompanying family members. Portability of an ATMP vs. transportation of the infant or juvenile patient to the manufacture site is an important factor for consideration in the design of a clinical trial. Trial sponsors should also consider whether there is a practical distribution strategy for the marketed product. This and other key factors to consider for efficient translation from research to routine therapy are described in Table 2.

Cost effective ATMP development for rare diseases is enabled by the orphan drug designation which offers market exclusivity once a product has marketing authorisation. The challenge to other emerging markets such as South and Central American countries is to forge links and form similar pan-continental regulatory frameworks. We would encourage an approach that mirrors either the FDA or EMEA to pave the way for the simplest international collaborations, multi-centre studies and eventually world-wide licencing.

Finally, of course the foremost question of cost versus benefit in the minds of patients, their families and their doctors is the potential for therapeutic benefit versus significant risk to life and/or quality of life. The ability to self-medicate, attend school, and associate with peers are key issues for children with disease and impact greatly on their quality of life. Thus curative treatment can have substantial quality of life benefit over life-long medication. The majority of ATMPs discussed above offer a real prospect of curative treatment but with equally real possibilities of malignancy, infection or other toxicities. Therefore the ultimate answer to this question is extremely individual and will only be found in time and with the conduct of carefully regulated fully informed clinical studies.

## Figures and Tables

**Table 1:** What common technological advances are most needed to deliver ATMPs as routine medicines worldwide?

✓ More efficient bulk production of GMP grade vectors and animal-free cell culture media ✓ Flexible, high capacity, automated systems for cell selection, cell culture and gene transduction ✓ Improved low weight vialling precision to: o enable dispensing of lyophilised vector doses for in vivo administration without additional QA review o improve ex vivo manufacturing consistency when using lyophilised raw materials ✓ Greater and more stable quantities of starting cells — iPS may enable this. ✓ Transportation solutions for distribution of fragile, temperature sensitive cell products over large distances: o Improved cryopreservation and hypothermic preservation media o Sterile packaging for transportation of 2D and 3D cell cultures o Logistics & importation restrictions — can we adapt the BMT/organ transplantation models?

**Table 2:** What are the key questions to consider early in development of gene/cell therapy for efficient translation later?

• What **IS** the final ATMP product?	• Gene modified cells? The vector? The cell sheet?
• What will be the final **formulation** of the ATMP?	• Fresh? Frozen? Cell suspension? Lyophilised vector? • Single or multiple doses per manufacture?
• What are the minimum quality specification(s)?	• Number of cells? Size? Viability? Transduction efficiency? • Will the QC results be available before the treatment is administered?
• Can your R&D production method be scaled up?	• Cell flasks vs. cell factories • Availability of GMP grade raw materials • Automated aseptic processing e.g. sepax, COBE, cliniMACS, WAVE, prodigy
• What is the national/international manufacturing strategy i.e. hubs vs. single manufacturing centre?	• Need to consider the robustness of the ATMP for transportation, i.e. is it preferable to transport the ATMP or the patient? • Will the patient need to return to the manufacturing centre for follow-up and monitoring tests?

*Special considerations for autologous products:*	
• Is there a holding therapy between harvest and return?	• Where there is a holding therapy (e.g. enzyme replacement) it is simpler to control the quality of autologous products. e.g. transduce the cells, freeze the product, check the quality i.e. transduction efficiency and sterility, then defrost and administer the product
• What if the product fails QA review?	• Can the raw material collection be repeated? • What is the alternative therapy for the patient?

**Table 3:** What are the key requirements to deliver a gene therapy service?

• MHRA licenced GMP manufacturing facility	I.e. ‘clean room’ min. 30 m^2^
• Contract with MHRA Qualified Person as named on that licence	E.g. 1 or more QPs employed as a consultant
• Co-ordination and preferably co-localisation of cell harvest, manipulation and cell culture laboratory facilities	E.g. ward/theatre, CD34+ cell selection lab & clean room on the same campus
• GMP grade (animal free) raw materials	EMEA or FDA GMP licenced suppliers
• Adequate appropriate cell source for therapy AND for validation of manufacturing methods	Validations need to be of a similar scale to the actual therapy
• Laboratories for microbiological, protein and genetic analysis	E.g. hospital diagnostics laboratories

**Table 4:** What are the practicalities of a GMP cell therapy manufacturing facility?

• *Infrastructure* — sponsoring institution, laboratories, offices, archive facilities
• Laboratory — with biological safety cabinets, isolators, *significant air handling*, staged changing areas
• Locked dedicated materials *storage* areas
• Real-time electronic *monitoring* of all the above facilities and equipment
• Regular preventative *maintenance*
• Dedicated daily *cleaning*
• Weekly and in-session microbiological *environmental monitoring*
• *Quality assurance* of equipment, assays and manufacturing method
• *Traceability* of all raw materials, and their method of manufacture
• *A team of highly trained personnel* — each step requires an ‘operator’ and a ‘checker’, each process requires QA review and QP release, hence a minimum of 3 manufacturing staff, plus a QA and a part-time QP
• Quality management system and extensive *documentation for GCP & GMP*
• Continuous corrective and preventative action (*CAPA*)
• Initial *ATMP licence* application and annual/bi-annual MHRA auditing
